# Global antibody response to *Staphylococcus aureus* live-cell vaccination

**DOI:** 10.1038/srep24754

**Published:** 2016-04-22

**Authors:** Martina Selle, Tobias Hertlein, Babett Oesterreich, Theresa Klemm, Peggy Kloppot, Elke Müller, Ralf Ehricht, Sebastian Stentzel, Barbara M. Bröker, Susanne Engelmann, Knut Ohlsen

**Affiliations:** 1 University Würzburg, Institute for Molecular Infection Biology, Würzburg, Germany; 2 University Greifswald, Institute for Microbiology, Greifswald, Germany; 3 Alere Technologies GmbH, Jena, Germany; 4 InfectoGnostics Research Campus Jena, Germany; 5 University Medicine Greifswald, Department of Immunology, Greifswald, Germany; 6 Technical University Braunschweig, Institute for Microbiology, Braunschweig, Germany; 7 Helmholtz-Zentrum für Infektionsforschung, Mikrobielle Proteomik, Braunschweig, Germany

## Abstract

The pathogen *Staphylococcus aureus* causes a broad range of severe diseases and is feared for its ability to rapidly develop resistance to antibiotic substances. The increasing number of highly resistant *S. aureus* infections has accelerated the search for alternative treatment options to close the widening gap in anti-*S. aureus* therapy. This study analyses the humoral immune response to vaccination of Balb/c mice with sublethal doses of live *S. aureus*. The elicited antibody pattern in the sera of intravenously and intramuscularly vaccinated mice was determined using of a recently developed protein array. We observed a specific antibody response against a broad set of *S. aureus* antigens which was stronger following i.v. than i.m. vaccination. Intravenous but not intramuscular vaccination protected mice against an intramuscular challenge infection with a high bacterial dose. Vaccine protection was correlated with the strength of the anti-*S. aureus* antibody response. This study identified novel vaccine candidates by using protein microarrays as an effective tool and showed that successful vaccination against *S. aureus* relies on the optimal route of administration.

*Staphylococcus aureus* is a gram-positive commensal prevalent on the skin and mucosa of mammals and birds. Nonetheless, it can elicit a broad range of severe diseases, including systemic infection, pneumonia and soft-tissue or skin infections^1^,^2^,^3^,^4^. The transformation of *S. aureus* from an asymptomatic colonizer to a life-threatening pathogen is characteristic of its ability to effectively adapt to changing environmental conditions.

In particular, the rapid development of antibiotic resistance has evolved into a global problem for healthcare systems. Methicillin-resistant strains (MRSA) are widely spread around the globe, being not only epidemic in hospitals but also in the community and in livestock^5^,^6^. It has been estimated that in 2011 up to 53 million people were colonized by MRSA^7^. The increasing number of severe MRSA infections causes enormous costs to healthcare systems and jeopardises effective treatments in modern medicine^6^,^7^. This highlights the urgency of early identification, appropriate treatment and vaccination against *S. aureus*. Currently, treatment options for MRSA infections include linezolid, daptomycin, telavancin, ceftaroline and ceftobiprole. Despite these options, the treatment failure rate of *S. aureus* infections remains high. Therefore, it is generally accepted that antibiotics alone cannot solve the overall therapeutic dilemma and other treatment modalities, such as vaccines or immunotherapies, are urgently needed.

Active immunization strategies are based on the capability of the adaptive immune system to develop immunological memory via specific immune cells and antibodies. It was hypothesized that the individual antibody profile in humans has an impact on the clinical outcome in patients^8^,^9^. This hypothesis is supported by the observation that immunoglobulin-deficient patients have a significantly increased risk of *S. aureus* infections^10^,^11^. Despite intensive research, a protective vaccine against *S. aureus* infection remains to be developed^12^,^13^.

In recent vaccination studies, immunization strategies focused either on surface structures of *S. aureus* such as capsule polysaccharides type 5 and 8, biofilm-associated poly-N-acetylglucosamine (PNAG), lipoteichoic acids (LTA) or on proteins presented on the surface of the bacterial cell such as ClfA and IsdB^14^,^15^,^16^,^17^,^18^,^19^,^20^. Unfortunately, clinical studies in humans could not prove any protective effect^21^,^22^,^23^. Further vaccine studies were directed at protein candidates that are secreted, e.g. PVL, alpha-toxin, enterotoxin B, PSMs, IsaA, LytM, and Nuc^24^,^25^,^26^,^27^,^28^,^29^. Many of these potential targets received preclinical validation as targets for passive and/or active immunization and clinical studies started recently to evaluate the efficacy of anti-Hla antibodies in nosocomial pneumonia^30^.

The disappointing results of human trials carried out to date raise the question of whether it is generally possible to develop a protective immune response against *S. aureus* in humans. Moreover, crucial vaccination targets to mediate an adequate antibody response against *S. aureus* remain to be identified.

In this study, we analysed the antibody profile generated during live-cell vaccination using two different application routes, intravenous and intramuscular, in mice using a recently developed protein array^31^. Mice were immunized three times with sublethal doses of live *S. aureus* to induce a specific anti-*S. aureus* immune response. Antibody and cytokine profiles elicited by the vaccination procedure were monitored. After recovery from the mild vaccine-induced infections, mice were re-challenged with a high dose of living *S. aureus*, which was inoculated into the thigh muscle. The outcome of this severe infection was correlated with high antibody titres against a particular subset of proteins generated after i.v. vaccination which were not produced after i.m. vaccination.

## Results

### *S. aureus* live-cell vaccination induces IgM and all IgG subclasses

**Figure 1:**
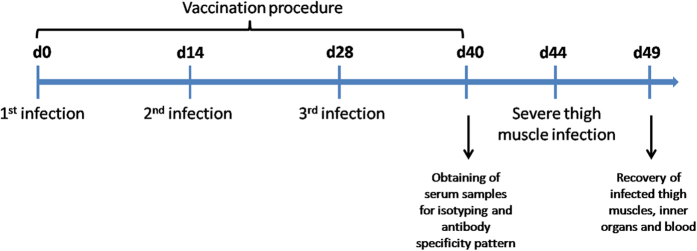
Time scale for repeated vaccination procedure and subsequent severe *S. aureus* challenge.

In order to analyse the humoral immune response after vaccination, mice were vaccinated three times with sublethal doses of live *S. aureus* Newman (2 × 10^6^ CFU), which were applied either intravenously (i.v.) or intramuscularly (i.m.) (Fig. 1).

**Figure 2:**
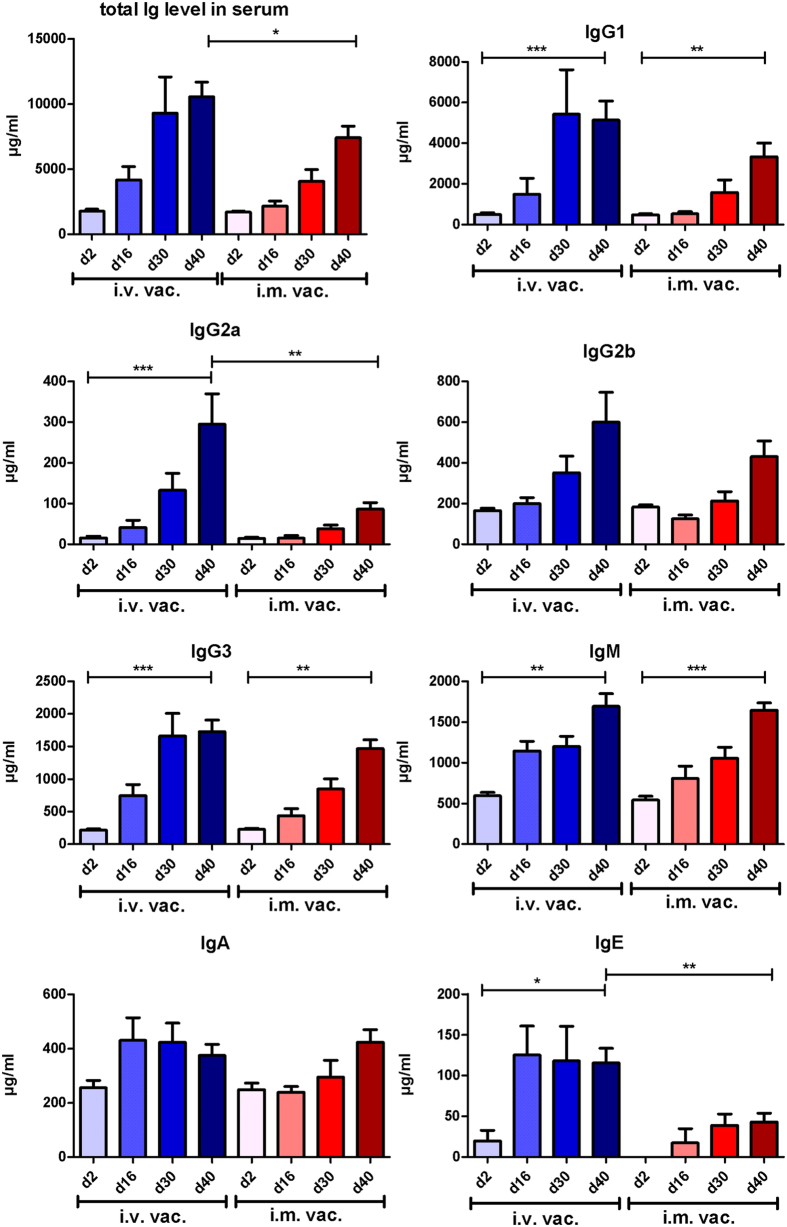
Total Ig level (IgG1, IgG2a, IgG2b, IgG3, IgM, IgA and IgE) and individual levels of IgG1, IgG2a, IgG2b, IgG3, IgM, IgA and IgE in the sera of intravenously (i.v. vac.) and intramuscularly (i.m. vac.) challenged mice at different time points during the procedure. Statistically significant differences were determined by one-way ANOVA and Mann-Whitney test, respectively, with a Bonferroni correction and are indicated by asterisks (*p < 0.05, **p < 0.01; ***p < 0.001).

Two days after each vaccination (d2, d16, d30) and 12 days after last vaccination (d40), serum was obtained and immunoglobulin serum concentrations and specificities were determined. We observed a continuous increase in total Ig serum concentrations in both i.v. and i.m. vaccinated mice (Fig. 2) indicating a robust anti-*S. aureus* specific antibody response induced by both immunization strategies. Intravenously vaccinated mice exhibited a sixfold Ig level increase from d2 to d40 [from 1761 +/− 406 μg/mL (d2) to 10544 +/− 3768 μg/mL (d40)]. Likewise, i.m. vaccinated mice showed a fourfold increase [from 1689 +/− 253 μg/mL (d2) to 7424 +/− 3009 μg/mL (d40)]. Although a strong increase in overall Ig levels was induced in both groups over time, the total Ig level was significantly lower in i.m. vaccinated mice compared with i.v. immunization. The maximal Ig concentrations were around 30% higher in i.v. vaccinated animals compared to i.m. immunization.

**Figure 3:**
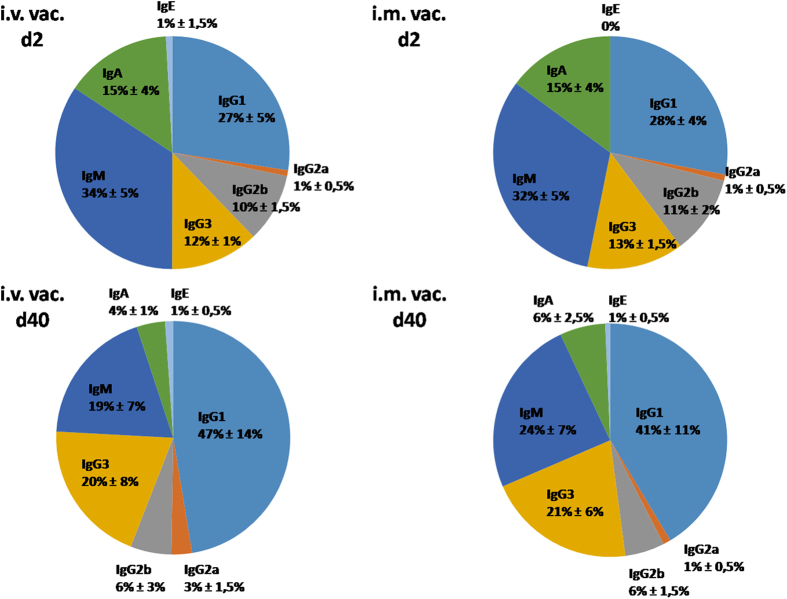
Percentage share of all tested Ig isotypes (IgG1, IgG2a, IgG2b, IgG3, IgM, IgA and IgE) at the beginning (d2) and end (d40) of the vaccination procedure in the sera of intravenously (i.v. vac.) and intramuscularly (i.m. vac.) vaccinated mice. Immunoglobulin levels were measured with flow cytometry-based bead assays in blood serum. Percentage share was calculated and illustrated with Microsoft Excel 2007 (Microsoft, USA).

A continuous rise in Ig concentration was observed for IgM and all IgG subclasses, while the levels of IgA and IgE exhibited only marginal changes during the vaccination process (Fig. 2). This shifted the composition of the antibody subclasses in the serum of the vaccinated mice: The relative concentrations of IgG1 and IgG3 increased in both models at the expense of IgM and IgA, while the proportion of IgG2a and IgG2b subclasses remained stable (Fig. 3).

### Antibody response to *S. aureus* vaccination depends on the application route

In order to characterize the antibody specificities as a response to vaccination, IgG antibody binding to different *S. aureus* antigens was determined using the Staph-Toxin-Array (Alere Technologies GmbH, Jena, Germany)^31^. This protein array is composed of 62 major antigens of *S. aureus* that were previously identified by global proteome analysis (antigens on the array are listed in Supplementary Table S1). The array technology facilitates the determination of antibody specificities in serum or other antibody-containing samples.

**Figure 4:**
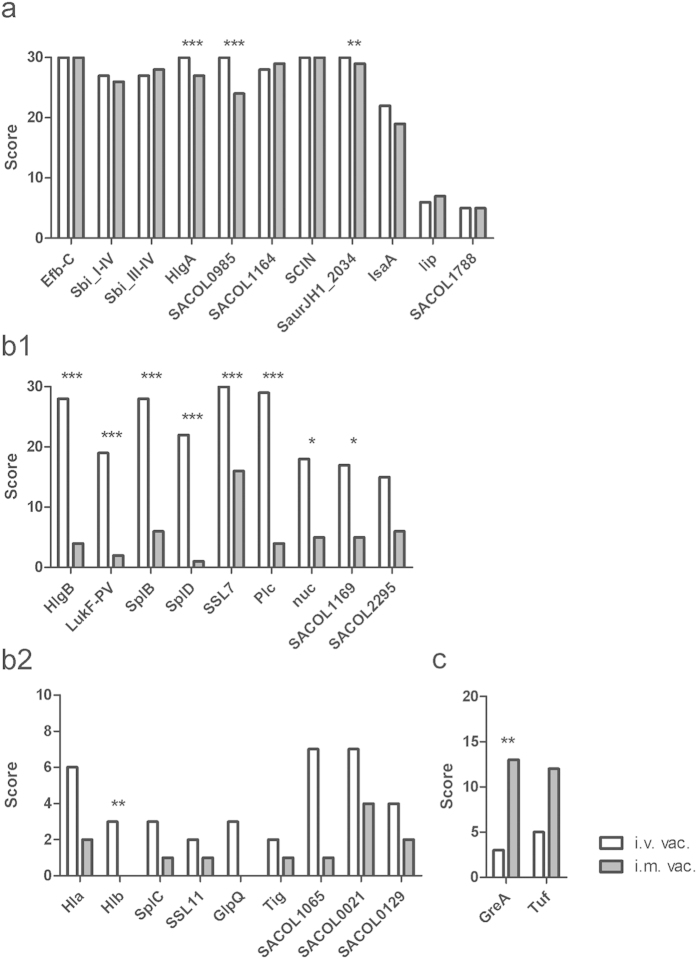
Antibody specificity pattern developed during vaccination with *S. aureus* Newman. Mice were vaccinated three times with sublethal doses of live *S. aureus* either intravenously (i.v. vac.) or into the thigh muscle (i.m. vac.) (n** **=** **10). Twelve days after the final vaccination (d40), sera were obtained and antibody specificities were determined using the Staph-Toxin-Array (Alere Technologies GmbH). Score values representing the antibody response in an entire group were calculated and are shown as bars. (**a**) antigens with high or similar antibody response in i.v. and i.m. vaccinated mice; (**b1** + **b2**) antigens with a stronger response in i.v. vac. mice; (**c**) antigens with a stronger response in i.m. vac. mice. Statistical significance of the differences between i.v. and i.m. vaccinated animals were tested using the Mann-Whitney test with Bonferroni correction and are indicated by asterisks (*p < 0.05, **p < 0.01; ***p < 0.001).

The strength of the antibody response was calculated based on relative signal intensities (AU), which were measured for each antigen and each mouse at two serum dilutions. By applying this method, we obtained values ranging from 0–8.5 AU, depending on the serum dilution. The background level was defined as 0.1 AU on the basis of previous validation of the array^31^. For each mouse, we calculated score values for each antigen according to following rule: a score value of 0 represented background signal intensity (≤0.1 AU), a score value of 1 represented measurements with low signal intensities (>0.1 to ≤1 AU), signal intensities between 1 AU and ≤4 AU were regarded as medium response and received a score value of 2, while all signal intensities above 4 AU were scored with 3 points. The sum of all score values of mice in one group resulted in the group score whose values ranged from 0 to 30 (N = 10 animals per group). These group score values are displayed in Fig. 4, while the signal intensities for each mouse are shown in Supplementary Fig. S1.

In total, we detected specific antibodies against 38 of the 62 tested antigens. No response was observed against 24 of the immobilized proteins, of which 6 proteins shared less than 40% sequence identity with *S. aureus* Newman, which was used for vaccination. The antibody response to all antigens is summarised in Supplementary Table S2.

Three different antibody response patterns were detected: antibodies against target antigens induced in both vaccination procedures, antibodies selectively induced in i.v. vaccinated mice and antibodies induced by i.m. vaccination. Eight antigens elicited a strong antibody response in all tested animals (Fig. 4a). Most are known virulence factors of *S. aureus* such as Efb (extracellular fibrinogen binding protein), HlgA (gamma-hemolysin), SCIN (staphylococcal complement inhibitor), SaurJH1_2034 (SCIN variant), SACOL1169 (SCIN-C variant), Sbi (second immunoglobulin binding protein), SACOL1164 (Efb homolog, extracellular complement binding protein [Ecb] or Efb homologous protein [Ehp]), SACOL0985 (extracellular adherence protein [Eap] homolog EapH2), and IsaA (immunodominant staphylococcal antigen A).

Intravenous vaccination triggered a strong antibody response against 15 antigens, which provoked no or only low levels of specific antibodies in the intramuscularly vaccinated mice (Fig. 4b). Among those were secreted virulence factors, such as the pore-forming toxins HlgB (gamma-toxin), and LukF-PV (leucocidin), Plc (phospholipase), serine proteases (SplD and SplB), the superantigen-like protein SSL7 and a fragment of Hlb (beta-hemolysin), Hlb is not expressed as functional toxin in strain Newman due to insertion of a prophage in *hlb*. Furthermore, we found significant differences in the antibody response to SACOL1169 (SCIN-C variant) and the extracellular enzyme Nuc (thermonuclease). Smaller differences in the antibody response between i.v. and i.m. vaccinated mice were observed for virulence factors such as Hla (alpha-toxin), GlpQ (glycerophosphoryl diester phosphodiesterase), SplC (serine protease) and SSL11 (superantigen-like protein). Additionally, we found minor differences for the cytosolic protein Tig (trigger factor) and for the as yet uncharacterised proteins SACOL2295, SACOL1065, SACOL0021, and SACOL0129.

On the other hand, there were only two antigens that raised a significantly higher antibody response in the thigh muscle vaccinated animals compared with intravenously vaccinated mice. These were the intracellular antigens GreA (transcription elongation factor) and Tuf (translation elongation factor) (Fig. 4c).

### Intravenous, but not intramuscular vaccination protects mice against challenge infection

**Figure 5:**
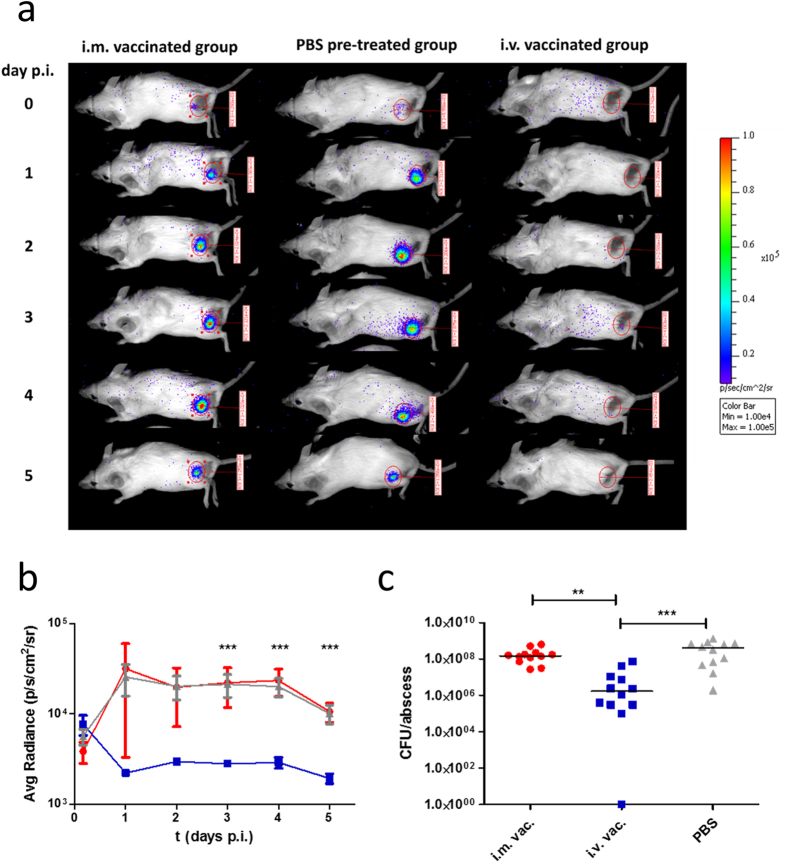
Effect of live-cell *S. aureus* vaccination on challenge infection in a thigh muscle abscess model. Bioluminescent *S. aureus* Newman *lux* (1 × 10^8^ CFU) was administered into the left thigh muscle of mice, which were previously vaccinated with viable *S. aureus* Newman intravenously (i.v. vac.), intramuscularly (i.m. vac.) or treated with PBS (PBS). (**a**) The expression of luciferase during infection resulted in photon emission. A bioluminescent signal was determined for each individual mouse in an oval region of interest at the site of infection by an *in vivo* imaging system (IVIS Lumina II, PerkinElmer, Waltham, MA, USA). One representative mouse is displayed for each group. (**b**) Bioluminescent signal for each individual mouse and mean (±SEM) bioluminescence values for each group were determined at indicated time points. (**c**) Abscessed tissue was removed 5 days p.i. and the number of CFU was determined after homogenization and plating. The median number and CFU values are shown. Statistically significant differences between the groups were determined by one way analysis of variance (ANOVA) with Dunn’s post test and are indicated by asterisks (**p < 0.01; ***p < 0.001).

Next, we were interested in whether mice that had been repeatedly vaccinated with a low dose of live bacteria would be protected against challenge infection with a high dose of *S. aureus*. Therefore, we challenged mice with strain Newman *lux* in the thigh muscle. *S. aureus* Newman *lux* is capable of autonomous luciferase expression due to a genome-encoded *luxABCDE* operon and serves as a bioluminescent reporter. Consequently, the course of infection can be visualized noninvasively by bioluminescence imaging. Photon emission was measured immediately after application of the luminescent *S. aureus* strain and then once a day until d5 p.i. (Fig. 5a). The initial bioluminescence signal was similar in all three groups of mice: naive, i.v. vaccinated and i.m. vaccinated animals. Twenty four hours p.i., photon emission decreased in the i.v. vaccinated group to almost background levels, while it strongly increased in both i.m. vaccinated and control mice. This difference was significant and remained stable for four days (Fig. 5b). Luminescence values decreased slightly at day 5 p.i. in all groups. However, the difference between the i.v. vaccinated group and the other two groups of mice remained significant.

In addition to *in vivo* imaging, we removed the abscessed tissue at day 5 p.i. to determine the colony forming units (CFU) per abscess (Fig. 5c). In correlation to the bioluminescence signal, the abscessed tissue of i.v. vaccinated mice harboured around 100-fold less viable *S. aureus* Newman *lux* cells than the infected tissue of the i.m. vaccinated or naïve control mice, a highly significant difference.

In order to determine whether *S. aureus* can persist after repeated vaccination with live bacteria, we examined the bacterial burden of inner organs (kidneys, liver, lung, heart and spleen), and thigh muscle tissue of i.m. vaccinated mice. Four mice per vaccination group were sacrificed 12 days after the last vaccination step and CFUs were determined by quantitative plating. No live bacteria were recovered from the thigh muscles, kidneys, livers or spleens, with the exception of one mouse in the i.v. vaccinated group, who had low numbers of bacteria in the kidneys (100 CFU in both kidneys), but no detectable bacteria in the other organs. This indicates that repeated vaccination with low doses of *S. aureus* Newman permitted complete eradication by the host immune system during the immunization process. Nonetheless, we vaccinated with wild-type Newman but challenged with genetically modified Newman *lux* in order to unambiguously distinguish between residual bacteria from the vaccination procedure and the severe challenge infection. Strain Newman *lux* harbours a kanamycin resistance cassette, which allowed distinguishing the vaccination strain from the challenge strain by plating organ homogenates on antibiotic-containing selection plates. All bacteria recovered from infected organs showed a kanamycin-resistant phenotype confirming the presence of the challenge strain *S. aureus* Newman *lux*. These findings confirm complete clearance of the administered bacteria prior to the challenge infection.

### Intravenous vaccination generates higher antibody levels than intramuscular vaccination during challenge infection

We were further interested in whether vaccination with live bacteria, either applied i.v. or i.m., would influence the strength and isotype pattern of the antibody response to the challenge infection. Interestingly, we found an additional strong increase in total Ig level in i.v. vaccinated mice after challenge infection compared to Ig levels after prior vaccination (d40). Especially, the amount of IgG1 and IgG2a raised strongly (IgG1: threefold, IgG2a: > twofold) (see Supplementary Fig. S2). The observed increase of IgG1 and IgG2a in i.v. vaccinated mice after the challenge infection was also reflected in a strong shift in isotype proportions to IgG1 (47% vs. 67% of the total Ig), and a concomitant decrease in IgG3 (20% vs. 12%) and, as expected, IgM (19% vs. 7%). All results concerning antibody isotypes after the challenge infection are summarized in Supplemetary Figs S2 and S3. These results indicate that the quantity as well as the quality of the antibody response, as reflected by the Ig subclass composition, is influenced by previous contact with *S. aureus*. Systemic application of live bacteria elicited a much stronger response than local inoculation.

### *S. aureus* induces T-cell cytokine response after i.m. vaccination

**Figure 6:**
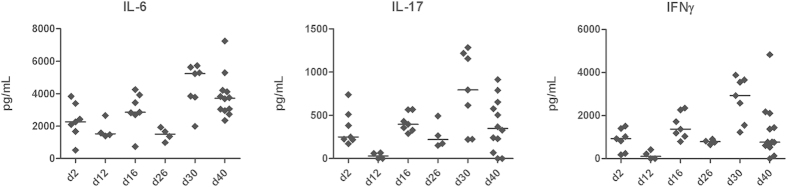
Cytokine levels of IL6, IL-17 and IFNγ at the site of infection of i.m. vaccinated mice. Abscessed tissue was removed at different time points during vaccination procedure and homogenised in sterile PBS. Cytokine levels were determined using the Mouse Th1/Th2/Th17/Th22 13plex Flow Cytomix Kit (eBioscience, Frankfurt am Main, Germany) and calculated using the corresponding software. Individual cytokine levels and corresponding median are shown in pg/mL organ homogenate.

We were further interested how cytokine response is induced during vaccination with live *S. aureus*. We analysed the production of typical T-cell associated cytokines such as IL-6, IL-17 and IFNγ at the primary site of infection in the abscessed tissue of the thigh muscle. For this, samples were taken two days (d2, d16, d30) and 12 days (d12, d26, d40), respectively after each i.m. vaccination step. Cytokine levels for all tested cytokines were significantly increased 2 days after each vaccination step (Fig. 6). In addition, we determined a constant raise of cytokine release at the site of infection after each vaccination step. Levels for IL-6 were 30% higher after the second and 130% higher after the third vaccination step compared to first vaccination. A similar increase was observed for IL-17 and IFNγ characterised by 220% increase after the third vaccination step. High levels of IL-17 and IL-6 are characteristic for a Th17 response^32^,^33^.

## Discussion

The aim of our work was to characterize the humoral immune response following vaccination of mice with live *S. aureus* by different routes and to identify putative vaccine candidates. For the analysis, we used a recently developed protein array, which covers 62 proteins of *S. aureus*, including several virulence factors such as cytolytic toxins and immune regulatory factors^31^. All mice developed a robust antibody response after repeated vaccination with low doses of live *S. aureus*. However, there was a significant difference in the antibody pattern depending on the route of vaccination. In general, i.v. vaccinated mice developed higher antibody titres and a broader spectrum of specificities compared with i.m. vaccinated animals. In particular, a strong response to known virulence factors, such as gamma-hemolysin Hlg, leukocidin Luk-PV, serine proteases SplB, SplD, and α-toxin Hla was in particular provoked in i.v. exposed animals. Hence, these antigens were notably produced during systemic exposure and were probably less present in muscle infection. The strength of the antibody response induced by vaccination with low doses of live *S. aureus* cells was later correlated with the level of protection in a severe challenge infection. Interestingly, i.m. vaccination did not protect against re-infection in the same organ. The lion’s share of community-associated *S. aureus* infections are infections affecting skin and soft tissues^34^,^35^. Evidently, the antibody specificity pattern and amount of antibodies that develop during this type of disease is not sufficient to overcome a later severe infection. Obviously, irrespective of putative differences in the quality of the immune response elicited by systemic versus local vaccination strategies, the intensity of the vaccine-induced immune response appeared to be decisive for protection. In a next step, individual antigens can be selected based on the presented results and used for a combinatorial vaccine to define the most protective antigens. Besides known vaccine candidates that were confirmed in our work including SCIN, Efb, IsaA, and Hla, several novel candidates merit further evaluation such as SACOL0985, SACOL2295, SACOL1065, SACOL0021, and SACOL0129. SACOL0985 was recently identified as Eap homologue named EapH2, which plays a role in immune evasion by blocking neutrophil serine proteases^36^. However, neither the biological function of the other novel candidate antigens is currently known nor their expression in other disease types than sepsis and wound infection.

There is an ongoing debate about the role of antibodies in immune defence against *S. aureus*^37^,^38^,^39^,^40^,^41^. Moreover, since none of the active and passive vaccination trials conducted to date has been met with success, fundamental scepticism about *S. aureus* vaccine programs has arisen. However, recent studies in *S. aureus* blood stream infection patients delivered evidence that high titres of *S. aureus*-specific antibodies are associated with less severe disease^8^,^9^,^31^. Likewise, *S. aureus* carriers possess significantly higher levels and a broader spectrum of antibodies than non-carriers^42^,^43^. It is assumed that this may be a reason for their better prognosis in life-threatening systemic infections^44^. Moreover, it has been reported by van der Kooi-Pol *et al*. that epidermolysis bullosa (EB) patients who are highly susceptible for skin infections produce high level of antibodies due to continuous exposure to *S. aureus* and its antigens but do not develop severe invasive infections. The authors concluded that high antibody titres may be responsible for increased protection against severe *S. aureus* infections. Interestingly, EB patients produce antibodies against a range of antigens also identified in this study such as IsaA, SCIN, HlgA, and Nuc^45^. However, it is still unclear whether the antibodies themselves mediate the protection and if so, which specificities are decisive.

The role of antibodies during antibacterial immune response is manifold, most prominently they can opsonize bacteria for phagocytosis and neutralize virulence factors that are released by the bacteria. In addition, they may be considered markers of an adaptive memory response, which also comprises *S. aureus*-reactive memory T cells^46^,^47^. Recently, it has been reported that in 95 to 100% of bacteremia patients the IgG response is significantly induced targeting several antigens including the immune modulators staphylococcal superantigen-like protein 3 (SSL3), SSL10, the immune evasion protein SCIN, the toxins γ-hemolysin (HlgB) and leukocidin F (LukF), a putative ABC transporter SA0688, the membrane-associated foldase PrsA and the surface bound and extracellular enzyme IsaA^48^. These findings are supported by a study of Bröker and co-workers, where a subset of pre-existing anti-*S. aureus* antibodies was correlated with severity of infection and disease progression. Patients with better outcome had significantly higher Ig levels directed against the extracellular enzymes IsaA, Plc and GlpQ, as well as the Eap homologue EapH2 (SACOL0985)^8^. Importantly, in our study, i.v. vaccinated mice produced high levels of antibodies against many of the antigens that also elicit IgG binding in bacteremia patients, including SCIN, LukF, Plc, GlpQ, SACOL0985 (EapH2), and the immunodominant antigen IsaA. The latter one has been shown to serve as promising target for antibody therapy^49^,^50^. These findings indicate expression of these virulence factors *in vivo* in both mice and humans. Our results are also in line with prior studies showing a difference between the IgG profiles of mice with lung infections and those with skin infections^51^. Overall, there is a substantial overlap between the IgG immune responses described in sepsis patients, EB patients, and i.v. vaccinated mice. This observation underscores the universality of expression of major virulence factors that may contribute to staphylococcal diseases in humans and also in mouse models. Nevertheless, there are a range of human-specific immune modulators such as SCIN, SCIN variants, CHIPS, superantigens (e.g. staphylococcal enterotoxin B or C, toxic shock syndrome toxin-1), or PVL which are either inactive or much less active in mice. This drawback in preclinical development may be compensated by including other animal species such as rabbits^52^.

Our data support the usefulness of mouse models for vaccine development despite limitations due to differentially expressed virulence factors and divergent immune responses to cytotoxins and superantigens^52^,^53^. This is in our view an important observation since the predictive power of currently used mouse models has been challenged as all of the vaccine candidates showed protective efficacy in preclinical mouse models but failed later in clinical trials^39^,^40^,^53^. However, the pure antibody response to virulence-associated factors is only one parameter, other immune defence mechanisms differ significantly between humans and mice. Probably, a higher prediction value can be reached when the validation of a rational designed vaccine includes animal studies in more than one species e.g. mice and rabbits combined with *in vitro* data with human cell types. However, the final proof of a human-specific vaccine can only be achieved in humans. In addition, the selection of relevant patient populations who can benefit from vaccination in clinical studies is a great challenge.

Furthermore, we analysed the isotype pattern following i.v. or i.m. infection in detail. There was a strong induction of all isotypes with exception of IgA and IgE after repeated challenge with viable *S. aureus*. The most abundant isotypes present at the beginning of vaccination were IgM and IgG1. In particular the IgG1 percentage increased dramatically in the i.v. model and also in the i.m. model from d2 to d40. Interestingly, the percentage of IgG3 increased also substantially in both models, reaching roughly 20% of all Igs. The IgG2a isotype represented a very low overall percentage in both vaccination routes, but tripled in the i.v. model from 1% to 3%. Almost unknown is the role of different isotypes in combating *S. aureus* infections. In a recent study, the impact of the isotype of an anti-staphylococcal enterotoxin B (SEB) antibody on binding, inflammatory response and *in vivo* protective ability was investigated^54^. In that study, Varshney *et al*. found that the IgG2a isotype variant exhibited significantly greater protection than IgG1 or IgG2b in murine SEB intoxication and *S. aureus* sepsis models. As expected, neither antigen specificity nor sensitivity was affected, but protection was associated with negative modulation of the inflammatory host response. Likewise, the IgG2a isotype of an antibody against *Bacillus anthracis* protective antigen was more efficient in preventing lethal toxicity than IgG2b, and IgG1 isotypes^55^. The authors claimed that the antibody isotype influences toxin neutralization efficacy through a mechanism that requires the engagement of FcγRs. These examples clearly illustrate the importance of isotypes, even for toxin neutralization showing that, besides interference with toxin binding to specific receptors, other antibody-mediated biological functions such as opsonisation may contribute to the anti-toxin effects.

In our study, IgG2a antibodies represented the lowest fraction of all isotypes, although this isotype is highly important for phagocytosis by neutrophils, and likely for toxin neutralization. In fact, IgG1 was the most abundant isotype which, in mice, plays a minor role in phagocytosis and complement activation by preferentially binding to the inhibitory FcγRIIb^56^,^57^. IgG subclass composition can dramatically influence the outcome of FcγR engagement by immune cells by directing the effector cells either in a pro- or anti-inflammatory state. Whereas IgG2a antibodies in mice have an overall activating function due to high-affinity binding to FcγRIV and low-affinity binding to FcγRIIb, IgG1 binds poorly to the activating FcγRIV. Consequently, the relative ratios of the bound IgG subclasses determines the net effect of leukocyte activities^57^. It can be speculated that the relatively high amount of the IgG1 subclass and low level of IgG2a reflects the shift into a more anti-inflammatory state at d40 post vaccination. This indicates a predominant Th2 response in vaccinated mice, which was shown previously to be typical for BALB/c mice^58^,^59^,^60^.

The cytokine patterns determined in this study indicate further an influence of vaccination on adaptive immune response especially on T-cells reflected by a fast and effective cytokine response to subsequent challenges with *S. aureus*. Vaccination induced all Th1, Th2, and Th17 T-cells, but it was not sufficient to limit severe *S. aureus* re-infection. In fact, our data are in line with former studies describing an important role of both humoral and cell-mediated immunity in skin and soft tissue infections^58^,^59^.

In conclusion, we have shown, as others have, that a vaccination approach against *S. aureus* infection is feasible, at least to attenuate or slow the progression of infection and identified novel putative vaccine candidates such as SACOL0985 (EapH2), SACOL1788, SACOL0021, and SACOL0129. The protection strongly correlates with a robust and diverse antibody response against a broad spectrum of *S. aureus* antigens, many of these belong to immune evasion protein families. Moreover, the route of immunization may be critical to the success of a vaccine in humans.

## Materials and Methods

### Bacterial strains

In this study, we have used *S. aureus* strains Newman and Newman *lux*. Strain Newman *lux* was obtained by transduction of the *luxABCDE* operon from *S. aureus* Xen29^61^. Strain Xen29 harbours a stabile copy of a modified *lux* operon from *Photorhabdus luminescencs* at a single integration site on the bacterial chromosome.

### Bacterial infection dose

*S. aureus* Newman and Newman *lux* were grown overnight in BHI medium (Oxoid, Wesel, Germany) under shaking conditions. Afterwards, they were diluted in fresh BHI and cultivated to mid-logarithmic phase. The bacterial cells were centrifuged (20 min, 4000 g) and taken up in BHI supplemented with 20% sterile glycerine. Prepared bacterial infection stocks were stored at −80 °C.

Prior to infection, bacteria were thawed at room temperature, washed and resuspended in sterile PBS to the desired concentration. The infection dose was adjusted according to the measured optical density of the culture. The infection dose was plated in appropriate dilutions on B agar plates to determine viable cell count.

### Murine vaccination model

Female BALB/c mice (18–20 g) were purchased from Charles River (Sulzfeld, Germany). All animals were kept in polypropylene cages and supplied with food and water *ad libitum*.

For vaccination, animals were infected three times with a sublethal dose of *S. aureus* Newman at intervals of 14 days. A group of ten mice was vaccinated in a murine kidney abscess model with 100 μl of *S. aureus* (2 × 10^6^ bacteria) intravenously via the tail vein. A second group of mice was inoculated in a thigh muscle infection model. Therefore the mice were shaved and 50 μl of a bacterial suspension (2 × 10^6^ bacteria) were injected into the right thigh muscle.

For analysis of the antibody pattern generated during the vaccination procedure, blood was obtained by heart puncture 12 days after the third infection interval. In reinfection studies, previously i.v. and i.m. vaccinated mice were re-infected with a high dose of *S. aureus*. Therefore, mice were infected 16 days after the last vaccination step with 1 × 10^8^ bacteria per mouse of *S. aureus* Newman *lux* in the thigh muscle.

### CFU enumeration

For CFU enumeration, organs were homogenised in 2 ml of sterile PBS using Dispomix (Bio-Budget Technologies GmbH, Krefeld, Germany). Appropriate serial dilutions were plated on mannitol-salt-phenol red agar plates for 24 h at 37 °C. Colony-forming units were counted and the bacterial burden was calculated as CFU per organ.

### Bioluminescence *in vivo* Imaging

For visualization of photon emission from the bioluminescent strain *S. aureus* Newman *lux*, an *in vivo* imaging system (IVIS Lumina II; Perkin Elmer, Waltham, MA, USA) was used. Mice were anesthetized with 2% isoflurane and imaged with following parameters: exposure, 120 s; F stop, 1; excitation, block; emission, open; field of view (FOV), D; and height, 1.5 cm. The bioluminescence signal was measured for a defined region of interest with standardized size for all mice and time points at the site of infection. The average radiance values were analysed using the corresponding Living Image 3.2 software (Perkin Elmer).

### Immunoglobulin isotyping

Immunoglobulin isotyping was performed using the mouse immunoglobulin isotyping panel FlowCytomix 6plex Kit and IgE FlowCytomix Simplex Kit obtained from eBioscience (Frankfurt am Main, Germany). Isotyping was performed according to the manufacturer’s protocol. The readout was made with a MACSQuant Analyzer (Miltenyi Biotec, Bergisch Gladbach, Germany) and immunoglobulin titres were calculated using FlowCytomix Pro Analysis software (eBioscience).

Further validation of IgG1 levels in serum samples was performed with the Mouse IgG1 ELISA Ready-SET-Go! (eBioscience). Isotyping was performed according to the manufacturer’s protocol. Immunoglobulin titres were calculated with GraphPad Prism (GraphPad, La Jolla, CA, USA).

### Determination of Cytokine levels

Cytokine levels were detected using the Mouse Th1/Th2/Th17/Th22 13plex Flow Cytomix Kit (eBioscience) according to the manufacturer’s protocol. Cytokine data were read by a MACSQuant Analyzer (Miltenyi Biotec) and data were analysed using FlowCytomix Pro Analysis software (eBioscience).

### Evaluation of the antibody response

The antibody specificity pattern was determined using the Staph-Toxin-Array. Staph-Toxin-Ag03 array strips and the detection substrate were provided by Alere Technologies GmbH (Jena, Germany). Every protein array harbours 63 different *S. aureus*-specific antigens which are covalently linked to the array surface. The *S. aureus*-specific antigens were immobilized in different concentrations ranging from 0.01–0.5 mg/ml^31^,^62^. Antibody detection was performed according to the manufacturer’s protocol using a HRP-conjugated anti-mouse IgG antibody (A3673, Sigma-Aldrich, Taufkirchen, Germany). The read-out was executed with an *ArrayMate* device and the corresponding software *Iconoclust* (Alere Technologies). A list of all immobilised *S. aureus*-specific antigens is shown in Supplementary Table S1. Additional information regarding recombinant expression and purification of spotted antigens are summarised in Kloppot *et al*.^31^ and in Supplementary Table S3-A/B/C in this work.

During the read out, relative (=AU) signal intensities of defined regions on the Staph-Toxin-Array were determined. Additionally, a score was developed to facilitate a grouping and the evaluation of the determined antibody response. Antibody response was classified for every antigen in the sera of ten mice. Relative signal intensities between 0.1 and 1 were interpreted as low antibody response, values between 1 and 4 as an intermediate response and a signal intensity higher than 4 was regarded as a high antibody response. From the derived signal intensity values we calculated an evaluation score according to the following equation:


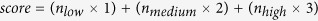


where *n*_*x*_ represents the number of mice with a low, medium or high antibody response, regarding the previous classification. We defined a low antibody response for an entire group of animals if the resulting score values ranged between 3 and 10, an intermediate response if the values ranged between 10 and 20 and a high antibody response if the score was higher than 20. The score value was used to assess the antibody response for a group of animals. A table of all defined thresholds and values necessary for the calculation of the score is shown in Supplementary Table S4.

### Statistical analysis

Analysis of variance (ANOVA) and the Mann-Whitney test with an additional Bonferroni correction were applied to evaluate differences in the antibody response for the specific antigens between different groups. All statistical analyses were performed using GraphPad Prism (GraphPad). For all tests performed, the significance level was set at a *P* value < 0.05.

### Ethics statement

All of the animal studies were approved by the local government of Franconia, Germany (approval number 2531.01-06/12) and performed in strict accordance with the guidelines for animal care and experimentation of German Animal Protection Law and the DIRECTIVE 2010/63/EU of the EU.

## Additional Information

**How to cite this article**: Selle, M. *et al*. Global antibody response to *Staphylococcus aureus* live-cell vaccination. *Sci. Rep.* **6**, 24754; doi: 10.1038/srep24754 (2016).

## Supplementary Material

Supplementary Information
