# Supplementary material for: Global antibody response to Staphylococcus aureus live-cell vaccination
Source: Sci Rep. 2016 Apr 22;6:24754. doi: 10.1038/srep24754 (PMC4840433; doi:10.1038/srep24754)
Supplement: Supplementary Information [file srep24754-s1.docx]

**Global antibody response to *Staphylococcus aureus* live-cell vaccination**

Martina Selle, Tobias Hertlein, Babett Oesterreich, Theresa Klemm, Peggy Kloppot, Elke Müller, Ralf Ehricht, Sebastian Stentzel, Barbara M. Bröker, Susanne Engelmann, Knut Ohlsen

Supplemetary Table S1: Overview of all *S. aureus* antigens immobilized on the Staph-Toxin-Ag03 classified ether by localization or known function. All proteins which are at least 98% identical in used *S. aureus* Newman strain are shown in bold.

Supplementary Table S2: Overview of detected antibody response against *S. aureus* specific antigens. If the antibody response was classified as high in at least one of the two tested groups, it is listed here as “high”.

Supplementary Table S3-A: Strains and plasmids used for recombinant expression of proteins immobilized on *Staph-Toxin-Array* 3^rd^ generation*.* For further antigens see Kloppot *et al.*

| **Strain** | **Reference** |
| --- | --- |
|  |  |
| ***E. coli* strains** |  |
| BL21 (DE3) pLysS | PMID: 3537305 |
| DH5α | PMID: 6345791 |
| SW 102 | PMID: 15731329 |
| Rosetta (DE-3) | Novagen, Merck, Darmstadt, Germany (http://wolfson.huji.ac.il/expression/rosetta.pdf) |
|  |  |
| ***Staphylococcus aureus* strains** |  |
| COL | PMID: 15774886 |
| N315 | PMID: 11418146 |
| Newman | PMID: 17951380 |
| RF122 | PMID: 12065548 |
| USA300_FPR3757 | PMID: 16517273 |
| H591 | PMID: 19112495 |
| JH1 | PMID:17517606 |
|  |  |
| **Plasmids** |  |
| pET-28a | Novagen, Merck, Darmstadt, Germany |
| pET101D TOPO | Life Technologies, Darmstadt, Germany |
| pET200/ D-TOPO | Life Technologies, Darmstadt, Germany |
| pPR-IBA1 | IBA GmbH, Goettingen, Germany |
| pPSG-IBA3 | IBA GmbH, Goettingen, Germany |
| pTXB1 | New England Biolabs, Frankfurt a.M., Germany |
| pFN18 | Promega, Mannheim, Germany |

**Supplementary Table S3-B: Oligonucleotides, plasmids and Enzymes used for recombinant expression of proteins immobilized on *Staph-Toxin-Array* 3^rd^ generation*.*** For further antigens see Kloppot *et al.* Proteins purified in the 1: Institute for Microbiology, Greifswald, Germany; 2: Institute for Molecular Infection Biology, Wuerzburg, Germany; 3: Institute for Immunology, Greifswald, Germany

| **Protein** | **Oligonucleotide 1 [*forward*]** | **Oligonucleotide 2 [*reverse*]** | **Restriction**  **enzyme** | **Plasmid** | **Strain for protein expression** | **Strain for Cloning** | ***tag*** |
| --- | --- | --- | --- | --- | --- | --- | --- |
| **HlgA1** | ATGGTAGGTCTCAAATGGAAAATAAGATAGAAGATATCGGCC | ATGGTAGGTCTCAGCGCTCTTAGGTGTGATGCTTTTAATTTTTAC | BsaI | pPR-IBA1 | BL21 (DE-3)  pLysS | DH5α | Strep,  C-terminal |
| **SACOL0021^1^** | ATGGTAGGTCTCAAATGCCTGATATTGCAAATGTCGACAATA | ATGGTAGGTCTCAGCGCTTTCAAGCCTCCCATCGTTATAAAC | BsaI | pPR-IBA1 | BL21 (DE-3)  pLysS | DH5α | Strep,  C-terminal |
| **SACOL0129^1^** | ATGGTAGGTCTCAAATGATAACGCATGAATCGCAACCTACA | ATGGTAGGTCTCAGCGCTTTTGAAGTCTGTTGTAATCAAGGTTT | BsaI | pPR-IBA1 | BL21 (DE-3)  pLysS | DH5α | Strep,  C-terminal |
| **SACOL0669^1^** | ATGGTAGGTCTCAAATGGCAAGTGGCAACTCTATTGATACT | ATGGTAGGTCTCAGCGCTTTTTTCCAAATCAATACGATATAATTTTA | BsaI | pPR-IBA1 | BL21 (DE-3)  pLysS | DH5α | Strep,  C-terminal |
| **SACOL07232** | CCCTCTAGAAATAATTTTGTTTAACTTTAAGAAGGAGATATACATATGTTTTTAACGCATCATGATGCAC | CGACTCGCCCTCGGGTAGGGCAACTAGTGCATCTCCCGTGATGCAATGGATGAATGCATAGCTAGAAAC | - | pTXB1 | Rosetta (DE-3) | SW102 | Tag free (Intein/CBD) |
| **SACOL0742^1^** | ATGGTAGGTCTCAAATGAAACGTATCAAACAACATCCGGAC | ATGGTAGGTCTCAGCGCTTGCCTCTGTATTGTTTTCTTTATTTTG | BsaI | pPR-IBA1 | BL21 (DE-3)  pLysS | DH5α | Strep,  C-terminal |
| **SACOL0755^1^** | ATGGTAGGTCTCAAATGAATGAAGATGCGCTTGCTAAGGTA | ATGGTAGGTCTCAGCGCTTTTCGTTATTATGCCTTGATTTGAAAT | BsaI | pPR-IBA1 | BL21 (DE-3)  pLysS | DH5α | Strep,  C-terminal |
| **SACOL0820^2^** | TTTTGCGATCGCCCAACAACATGGCACACAAGT | TTTTGTTTAAACTTAGTGGATGTAATTATATTTTC | SgfI, PmeI | pFN18 | BL21 (DE-3)  pLysS | DH5α | Tag free (Halo,  N-terminal) |
| **SACOL1065^1^** | ATGGTAGGTCTCAAATGAAAACGTCCCAAGATGCATTCGAA | ATGGTAGGTCTCAGCGCTATTTACAACACCATTTTGGTTATTTGA | BsaI | pPR-IBA1 | BL21 (DE-3)  pLysS | DH5α | Strep,  C-terminal |
| **SACOL1164^1^** | ATGGTAGGTCTCAAATGCAAACTAAAAACGTTGAAGCTGCTA | ATGGTAGGTCTCAGCGCTTTTTAAAGTATTATATTTTAAAACTAGATC | BsaI | pPR-IBA1 | BL21 (DE-3)  pLysS | DH5α | Strep,  C-terminal |
| **SACOL1870^1^** | ATGGTAGGTCTCAAATGATGAATACAAAATTTTTAGGTAAAACATT | ATGGTAGGTCTCAGCGCTATCAATTGTGATTTTGTTGATGAAGC | BsaI | pPR-IBA1 | BL21 (DE-3)  pLysS | DH5α | Strep,  C-terminal |
| **SACOL2179^1^** | ATGGTAGGTCTCAAATGAGTCAACGACAGGCACAAACATTT | ATGGTAGGTCTCAGCGCTTTTTTTCCATAAGAAGTCGATTAACTC | BsaI | pPR-IBA1 | BL21 (DE-3)  pLysS | DH5α | Strep,  C-terminal |
| **SACOL2295^1^** | ATGGTAGGTCTCAAATGGCTGAAAATTATACAAATTACAACAAC | ATGGTAGGTCTCAGCGCTAATATGGATGTAGTTGTAGTTACCTG | BsaI | pPR-IBA1 | BL21 (DE-3)  pLysS | DH5α | Strep,  C-terminal |
| **SACOL2661^2^** | GGTGGTCATATGATTTATGCTGCAATAAATCA | GGTGGTTGCTCTTCCGCAATTTTTAGGTGATTGTGCTT | NdeI, SapI | pTXB1 | Rosetta (DE-3) | DH5α | Tag free (Intein/CBD) |
| **SaurJH1_2034^2^** | CCCTCTAGAAATAATTTTGTTTAACTTTAAGAAGGAGATATACATATGTCACCACTAGTAACTAATCTAG | CGACTCGCCCTCGGGTAGGGCAACTAGTGCATCTCCCGTGATGCAATATTTACTTTTTAGTGCTTCGTC | - | pTXB1 | Rosetta (DE-3) | SW102 | Tag free (Intein/CBD) |
| **SplC1 (SAUSA300_1756)^3^** | ATGGTAGGTCTCAAATGGTCGTTGAAGAGACACAACAAATAG | ATGGTAGGTCTCAGCGCTTTGTTCAATGTGCTTTTGAATAAAATC | BsaI | pPR-IBA1 | BL21 (DE-3)  pLysS | DH5α | Strep,  C-terminal |
| **SplD^3^ (SAUSA300_1755)** | ATGGTAGGTCTCAAATGGAAAATAGTGTGAAATTAATTACCAAC | ATGGTAGGTCTCAGCGCTTTTATCTAAATTATCTGCAATAAATTTCT | BsaI | pPR-IBA1 | BL21 (DE-3)  pLysS | DH5α | Strep,  C-terminal |
| **SspA^1^** | ATGGTAGGTCTCAAATGTTATCATCAAAAGCTATGGACAATCA | ATGGTAGGTCTCAGCGCTTGCAGCGTCAGGGTTGTCTGAA | BsaI | pPR-IBA1 | BL21 (DE-3)  pLysS | DH5α | Strep,  C-terminal |

**Supplementary Table S3-C: Characteristics of recombinant expressed proteins immobilized on *Staph-Toxin-Array* 3^rd^ generation*.*** For further antigens see Kloppot *et al.*

| **Protein** | **Definition^[[1]](#footnote-1)^** | **Organism;**  **Taxon ID^1^** | **GI Number^1^** | **Predicted localisation^[[2]](#footnote-2)^** | **conserved/ variable^1^** |
| --- | --- | --- | --- | --- | --- |
| **SplC** | *serine protease SplC* | USA300_FPR3757; 451515 | 87160903 | Secreted (released) | conserved |
| **SplD** | *serine protease SplD* | USA300_FPR3757; 451515 | 87160514 | N-terminal anchored | variable |
| **SspA** | *serine protease; V8 protease; glutamyl endopeptidase* | N315; 158879 | 15926635 | Secreted (released) | conserved |
| **SACOL0021** | *yycH; hypothetical protein* | COL; 93062 | 57651129 | N-terminal anchored | conserved |
| **SACOL0129** | *hypothetical protein* | COL; 93062 | 57651150 | Secreted (released) | conserved |
| **SACOL0669** | *hypothetical protein* | COL; 93062 | 57650079 | Secreted (released) | conserved |
| **SACOL0723** | *LysM domain-containing protein* | COL; 93062 | 57651506 | Secreted (released) | conserved |
| **SACOL0742** | *hypothetical protein* | COL; 93062 | 57651523 | N-terminal anchored | conserved |
| **SACOL0755** | *hypothetical protein* | COL; 93062 | 57651536 | Secreted (released) | conserved |
| **SACOL0820** | *LysM domain-containing protein* | COL; 93062 | 57650096 | Secreted (released) | conserved |
| **SACOL1065** | *hypothetical protein* | COL; 93062 | 57650249 | Secreted (released) | conserved |
| **SACOL1164** | *fibrinogen binding-like protein, Ecb, Ehp* | COL; 93062 | 57650263 | Secreted (released) | conserved |
| **SACOL1870** | *hypothetical protein* | COL; 93062 | 57650605 | Secreted (released) | variable |
| **SACOL2179** | *hypothetical protein* | COL; 93062 | 57650775 | Secreted (released) | conserved |
| **SACOL2661** | *hypothetical protein* | COL; 93062 | 57652379 | Secreted (released) | variable |
| **SaurJH1_2034** | *hypothetical protein; staphylococcal complement inhibitor SCIN* | JH1; 359787 | 150394477 | Secreted (released) | variable |
| **HlgA** | *gamma-hemolysin, component A* | COL; 93062 | 57650963 | Secreted (released) | conserved |

Supplementary Table S4: Calculation of the score for grouping and evaluation of the antibody response for each individual mouse and for a group of ten mice.

Supplementary Figure S1: Antibody specificity pattern produced during vaccination with *S. aureus* Newman.
Mice were vaccinated three times with a sublethal dose (2x10^6^ CFU) of *S. aureus* either intravenously or into the thigh muscle (n=10). Ten days after the final vaccination, sera were obtained and antibody specificities were determined by using Staph-Toxin-Array. All signal intensities [AU] are shown in the scatter dot blots. Score values (see figure 2) were calculated on the basis of measured signal intensities illustrated in this figure. A: antigens with similar antibody response in i.v. and i.m. vaccinated mice; B1+B2: antigens with a stronger response in i.v. vac. mice; C: antigens with a stronger response in i.m. vac. mice.

Supplementary Figure S2: Levels of total Ig and immunoglobulin classes and subclasses in the serum of high dose intramuscularly infected animals at day 5 p.i.. Serum was recovered at the end point of the experiment and immunoglobulin levels measured with flow cytometry based bead assays and ELISAs. Data was analysed with FlowCytomix Pro software (eBioscience) and with GraphPad Prism 5.0 (GraphPad). Displayed are the means +/- SEM for each cytokine and group. Statistical significance was determined with Mann-Whitney U test and indicated by asterisks (*: P<0.05, **: P<0.01, ***: P<0.005).

Supplementary Figure S3: Comparison of immunoglobulin percentage shares in the blood serum after high dose intramuscular challenge with *S. aureus* for intravenously or intramuscularly vaccinated mice. Serum was recovered at the end point of the experiment and immunoglobulin levels measured with flow cytometry based bead assays and ELISAs. Percentage share was calculated and displayed with Microsoft Excel 2007 (Microsoft).

1. Allgemeine Datenbanken [↑](#footnote-ref-1)
2. *LocateP DataBase © 2007-2012 by Miaomiao Zhou* [↑](#footnote-ref-2)
